# Why Camera-Based and Scale-Based Measurements Differ: A Physiological Model of Diurnal Weight Variation in Finishing Pigs

**DOI:** 10.3390/ani16030498

**Published:** 2026-02-05

**Authors:** Kikuhito Kawasue, Khin Dagon Win, Tadaaki Tokunaga

**Affiliations:** 1 Faculty of Engineering, University of Miyazaki, Miyazaki 889-2192, Japan; 2 Faculty of Agriculture, University of Miyazaki, Miyazaki 889-2192, Japan; toku@miyazaki-u.ac.jp

**Keywords:** body weight fluctuation, camera-based estimation, feeding behavior, finishing pigs, precision livestock farming, simulation model, water intake, weight measurement accuracy

## Abstract

Live weight is widely used as a reference indicator for growth performance and for evaluating the accuracy of weight measurement technologies in pig production. However, live weight is not a fixed physiological quantity, and finishing pigs naturally experience substantial short-term mass fluctuations due to normal behaviors such as drinking, feeding, urination, and defecation. In this study, we integrated published physiological and behavioral parameters into a stochastic simulation model to quantify within-day live-weight dynamics in finishing pigs weighing approximately 100 kg. The simulation was conducted with 1-min temporal resolution over a 24-h period. The model demonstrated that short-term weight fluctuations of approximately ±3–5 kg can occur within a single day, even when measurement error is minimal. Across 1000 simulated pigs, the mean daily fluctuation range was 4.2 kg, confirming that kilogram-scale variation is physiologically expected under normal conditions. These results provide a plausible physiological basis for understanding the frequently reported discrepancies between camera-based weight estimates and instantaneous floor-scale measurements. Camera systems primarily reflect body mass derived from external morphology, whereas floor scales measure instantaneous total mass that includes transient contributions from gastrointestinal contents, ingested water, and retained waste. Consequently, direct comparisons based on instantaneous scale readings can be misleading when used as ground truth. Our findings indicate that commonly cited accuracy claims of ±2–3 kg for camera weighing systems should be interpreted with caution, as normal physiological weight variation often exceeds this range. Recognizing live weight as a dynamic physiological variable is essential for developing biologically meaningful evaluation frameworks and for the appropriate interpretation and comparison of weight measurement technologies in precision livestock farming.

## Simple Summary

Live weight is widely used to assess pig growth and to evaluate the performance of weighing technologies. However, pig body weight is not constant throughout the day. Normal activities such as drinking, eating, urinating, and defecating naturally change the amount of water and digesta within the body, leading to short-term weight fluctuations. A key issue addressed in this study is that these normal physiological variations are often overlooked when discrepancies are observed between camera-based weight estimates, which infer weight from body shape, and floor-scale measurements. The aim of this study was to quantify the extent to which pig body weight can vary within a single day solely due to normal physiological processes. Using published data, we developed a simulation model to reproduce diurnal weight changes in finishing pigs weighing approximately 100 kg. These findings may help explain why camera-based measurements often differ from scale readings by several kilograms. Such differences do not necessarily indicate measurement errors but instead reflect normal biological variation. Recognizing body weight as a dynamic physiological variable is essential for fair evaluation of weighing technologies and improved decision-making in modern pig production.

## 1. Introduction

Monitoring live weight is essential for evaluating growth performance, optimizing feeding strategies, and determining appropriate market timing in pig production. In recent years, camera-based weighing systems have emerged as promising non-contact alternatives to conventional floor scales within the framework of precision livestock farming. These systems estimate body mass from external morphology and enable frequent measurements without disturbing the animals. However, discrepancies of several kilograms are often observed between camera-based estimates and scale-based measurements. Such discrepancies have been repeatedly reported in studies using camera-based weighing systems but are still commonly interpreted primarily as measurement errors, even though these discrepancies may also reflect normal physiological body mass fluctuations [1,2]. Under well-controlled acquisition protocols, depth-camera-based weighing systems have reported root mean square errors of approximately 1.5–2.0 kg when validated against scale-based body weight measurements in commercial pig farms, representing upper-bound performance achieved under favorable measurement conditions [3].

However, it is important to recognize that performance reported under controlled or optimized acquisition protocols may not directly translate to routine commercial conditions. In practical farm environments, camera-based phenotyping systems must operate under variable illumination, occlusion due to animal interactions and housing structures, heterogeneous viewpoints and distances, and occasional sensor contamination or vibration; these factors can systematically influence both measurement quality and the validity of scale-based comparisons. Recent vision-based livestock monitoring studies conducted under real production conditions highlight the need to explicitly account for such deployment constraints when interpreting system accuracy and evaluation results [4].

In Japan, official carcass grading criteria specify narrow acceptable carcass weight ranges to obtain higher grades. For example [5], the Japan Meat Grading Association (JMGA) defines the “Upper” grade weight ranges as 68–83 kg for skinned carcasses and 74–89 kg for scalded carcasses. As a result, weight measurement accuracy at the farm level must be sufficient to determine whether an animal lies within such ranges. Within-day weight fluctuations can shift animals across carcass/marketing thresholds, leading to suboptimal shipment timing and avoidable profit loss. If short-term weight changes are misinterpreted as poor growth, feeding may be adjusted unnecessarily, increasing feed use and cost without improving true performance. Under these conditions, daily physiological weight fluctuations can become comparable to the tolerance required for grading-related decisions, potentially leading to apparent discrepancies between different measurement technologies or measurement time points.

Importantly, live weight as measured by a floor scale is not a stable physiological quantity but instead fluctuates continuously throughout the day. These fluctuations arise from normal behaviors such as drinking, feeding, urination, and defecation, as well as changes in the amount of water and digesta retained within the body. Previous studies have investigated individual components of these processes, including drinking frequency and water intake patterns [6,7,8,9,10], voluntary feed intake and feeding behavior [11,12,13], and gastrointestinal fill and digestive passage dynamics [14,15,16]. Although some studies have reported short-term fluctuations in body weight or related physiological processes [13,14,15,16,17], these findings remain fragmented, and the combined effects of daily behaviors on within-day body-weight variation have rarely been integrated into a unified physiological framework. More comprehensive modelling approaches addressing pig growth dynamics have also been reviewed in the literature [18], but these frameworks do not explicitly focus on short-term physiological weight fluctuations within a single day.

To our knowledge, no previous study has quantitatively integrated published data on drinking, feeding, urination, and defecation to model how these processes collectively generate within-day fluctuations in body mass in finishing pigs. More critically, this inherent physiological variability has not been explicitly considered in the evaluation of camera-based weight estimation systems, despite frequent reports of multi-kilogram differences between camera-based weight estimates and instantaneous scale measurements [1,2,3]. Without accounting for normal physiological dynamics, such differences may be mistakenly attributed to measurement inaccuracies. Although weighing at a fixed time can reduce variability within a single farm, a universal cross-farm setting cannot assume identical weighing times or identical timing relative to feeding and drinking events; therefore, within-day physiological fluctuation becomes an unavoidable source of variation in reference weights.

In this study, we integrated established behavioral and physiological parameters from the literature [6,7,8,9,10,11,12,13,14,15,16,17,18] to construct a stochastic simulation of daily body-weight dynamics in finishing pigs. The objective of this study was to quantify the magnitude of physiologically expected short-term weight fluctuations and to clarify their implications for interpreting the accuracy and limitations of camera-based weighing technologies used in pig production. This framework is parameterized for finishing pigs weighing approximately 100 kg, and the magnitude of within-day fluctuations may vary with growth stage, environmental conditions, and management practices.

## 2. Methods

### 2.1. Overview of the Modelling Approach

A stochastic simulation model was developed to reproduce short-term, within-day fluctuations in the live weight of finishing pigs weighing approximately 100 kg. The model represents live weight as the sum of multiple transient mass components associated with normal daily behaviors, including drinking, feeding, urination, and defecation. All processes were treated as probabilistic events occurring over a 24 h period, and each event produced either a positive or negative contribution to body mass. Parameter ranges were selected to reflect physiologically plausible orders of magnitude reported in previous studies and veterinary practice.

The objective of the model was not to predict individual animal behavior, but rather to quantify physiologically plausible short-term variability in live weight under normal production conditions.

For simplicity and transparency, several physiological processes were modeled using linear clearance or passage assumptions, including linear decay of drinking-related water mass and linear gastrointestinal transit over a fixed time window. While these assumptions are appropriate for a Communication and for capturing order-of-magnitude effects, they oversimplify real biological kinetics, which can be nonlinear, state-dependent, and influenced by circadian rhythms. Because the interpretive conclusions of this study depend on the magnitude of within-day weight fluctuations, we explicitly examined the sensitivity of the results to key parameters such as drinking clearance time and gastrointestinal transit time, as reported in the one-factor-at-a-time sensitivity analysis (Supplementary Figure S1).

All model parameters, distributions, and fixed constants used in the simulations are summarized in Table S1 in the Supplementary Materials. The Supplementary Materials were calculated using Anaconda (Anaconda, Inc., Austin, TX, USA) with Python 3.10.

### 2.2. Drinking Behavior and Water Intake

Drinking behavior was modeled as a sequence of discrete drinking events distributed across the day. The number of drinking bouts per day was sampled from a Poisson distribution constrained to a physiologically realistic range (8–15 visits per day). Event times were assigned with a higher probability during daytime active periods (06:00–22:00) and reduced probability during night hours.

Each drinking event produced an instantaneous increase in body mass corresponding to the consumed water volume. Event magnitudes were sampled from a gamma distribution and limited to a realistic range (approximately 0.4–1.6 kg per bout). Following each event, the ingested water mass was assumed to be cleared linearly over a fixed physiological time window, representing redistribution and elimination processes. In the present implementation, the drinking-related mass contribution was assumed to decay linearly over a fixed clearance window of 180 min. This linear clearance produced a gradual decrease in the water-related mass contribution between drinking events.

### 2.3. Feeding Events and Gastrointestinal Content

Daily feed intake was set within typical values for finishing pigs (2.5–3.0 kg per day). Feeding was represented as a small number of discrete meals (3–6 meals per day), with meal sizes sampled from a Dirichlet distribution to preserve realistic variability among feeding events.

Each feeding event produced an instantaneous increase in gastrointestinal mass equal to the ingested feed plus an associated water binding component. Gastrointestinal content was subsequently reduced using a linear passage model with a fixed transit time of 12 h, representing digestion and movement through the gastrointestinal tract. A 15% water-binding component relative to ingested feed mass was included.

### 2.4. Urination and Defecation

Urination and defecation were modelled as reservoir-type processes. Urine and feces masses accumulated continuously over time at constant rates corresponding to daily excretion totals. Elimination events occurred at discrete times, sampled from physiologically plausible ranges (6–10 urinations per day and 2–4 defecations per day).

At each elimination event, an event-specific amount of mass (sampled independently for each event) was removed instantaneously from the corresponding reservoir. This approach generated stepwise decreases in live weight at elimination times and gradual increases between events, reflecting normal physiological accumulation processes.

### 2.5. Integration of Mass Components and Daily Growth Constraint

At each simulation time step (1-min resolution), live weight was computed as the sum of all mass components:

W(t) = W0 + Wwater(t) + WGI(t) + Wurine(t) + Wfeces(t),

where W0 denotes baseline body mass, Wwater(t) the water-related mass component, WGI(t) the gastrointestinal content mass, Wurine(t) the urine-related mass component, and Wfeces(t) the fecal mass component.

To reflect normal growth, a linear daily growth constraint was applied such that the net change in live weight over the 24-h period equalled +0.8 kg. This adjustment ensured that simulated within-day fluctuations were evaluated relative to a realistic daily growth trajectory while preserving the temporal structure of short-term variability.

### 2.6. Simulation Outputs

For each simulated pig, the model produced a continuous 24-h trajectory of live weight. A total of 1000 virtual pigs were simulated to capture variability arising from stochastic behavioral patterns.

The following output measures were calculated:

1. Within-day fluctuation range (maximum minus minimum live weight),
2. Short-term deviations over 1–2 h intervals,
3. Magnitudes of transient positive and negative weight changes,
4. Distribution of fluctuation amplitudes across the simulated population.

These outputs were used to evaluate whether kilogram-scale discrepancies between instantaneous scale measurements and camera-based weight estimates could arise solely from normal physiological variation.

## 3. Results

**Figure 1:**
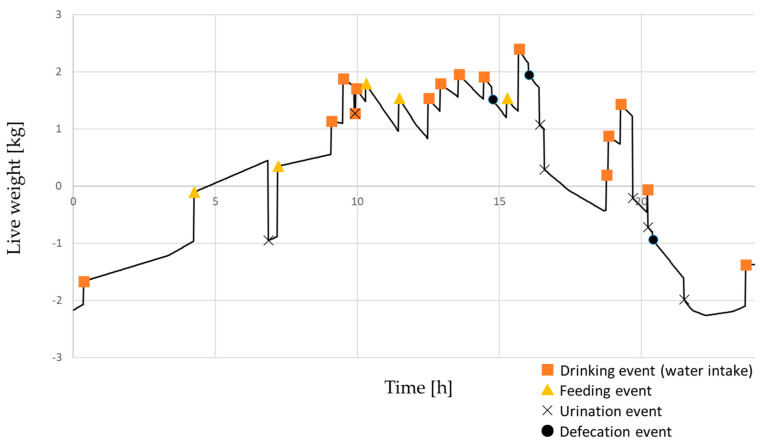
Representative 24-h trajectory of simulated change in live weight (relative to baseline) in a 100-kg finishing pig, showing within-day fluctuations superimposed on a net daily gain of 0.8 kg. Smooth recovery segments during night hours reflect simplified linear accumulation and clearance assumptions and do not represent detailed physiological dynamics.

Figure 1 illustrates a representative 24-h trajectory of simulated change in live weight (relative to baseline) in a 100-kg finishing pig. Normal drinking, feeding, urination, and defecation events generate an oscillating weight pattern, resulting in a within-day fluctuation range of approximately 3–5 kg, even in the absence of substantial measurement error. Across 1000 simulated finishing pigs, the mean daily fluctuation range (maximum minus minimum live weight) was 4.2 kg, with an interquartile range of 3.4–5.1 kg. These results confirm that short-term weight variations of several kilograms are physiologically expected under normal conditions. Drinking and urination events produced the largest transient changes in body mass, while feeding contributed more gradual increases followed by declines associated with gastrointestinal passage.

When the simulated trajectories were compared with commonly reported accuracy claims (±2–3 kg) for commercial camera-based weighing systems, a large proportion of within-day fluctuations exceeded this range at some point during the day. This demonstrates that differences of several kilograms between camera-based estimates and instantaneous scale measurements can arise even when both systems operate ideally, reflecting the dynamic physiological nature of live weight rather than measurement inaccuracy.

A one-factor-at-a-time sensitivity analysis was conducted by perturbing key parameters related to drinking clearance, gastrointestinal transit, and urination event magnitude (Supplementary Figure S1). Across all tested settings, the within-day fluctuation range consistently remained in the kilogram scale, and the main conclusion was unchanged.

## 4. Discussion

This study shows that finishing pigs exhibit substantial short-term fluctuations in live weight as a normal consequence of daily physiological processes, including drinking, feeding, urination, and defecation. The stochastic simulations indicate that within-day weight variations of approximately ±3–5 kg are not exceptional but physiologically expected in pigs weighing around 100 kg. Importantly, these fluctuations can arise independently of measurement error, highlighting that live weight itself is an inherently dynamic physiological quantity rather than a fixed reference value.

Among the simulated mass components, short-term fluctuations are primarily dominated by water intake and excretion events, whereas gastrointestinal contents contribute to variations on a several-hour timescale. Growth-related mass changes mainly affect longer-term trends and do not explain short-term discrepancies between camera-based and scale measurements.

These findings have direct implications for how weight measurement accuracy is interpreted in precision livestock farming. Camera-based weighing systems estimate body mass primarily from external morphology and therefore tend to reflect the relatively stable structural component of the animal’s body. In contrast, floor scales measure instantaneous total mass, which includes transient contributions from gastrointestinal contents, ingested water, and retained urine and feces. As these internal components can change by several kilograms within short time intervals, discrepancies between camera-based estimates and scale measurements are a natural outcome of physiological dynamics rather than necessarily an indication of poor measurement performance. This issue is amplified in cross-farm applications because reference weights are commonly collected at different times of day and under different routines, so several-kilogram discrepancies can reflect normal physiology rather than sensor or model error.

Although the present model is parameterized for finishing pigs weighing approximately 100 kg, the magnitude and temporal structure of within-day weight fluctuations are expected to differ under other conditions. In lighter pigs, absolute intake and excretion events are typically smaller, whereas under heat stress or alternative feeding regimes, drinking frequency and excretion patterns may be altered, potentially modifying fluctuation amplitude. These factors may influence the quantitative range of fluctuations but do not change the fundamental conclusion that live weight is an inherently dynamic physiological variable.

Our results suggest that commonly reported accuracy claims of ±2–3 kg for camera-based weighing systems should be interpreted with caution. When the true live weight of an animal fluctuates beyond this range during normal daily activity, direct comparisons between camera-based estimates and instantaneous scale readings become conceptually problematic. Under such conditions, deviations of several kilograms may reflect the timing of measurement relative to behavioral events rather than technical limitations of the measurement system. This observation may help explain why camera-based systems are sometimes reported to “overestimate” or “underestimate” body weight when benchmarked against floor scales.

An important implication of these findings concerns the standardized measurement timing of reference (floor-scale) measurements in practical settings. In commercial pig farms, floor-scale measurements are often conducted at different times of day depending on farm routines and operator availability, such as in the morning, midday, or late afternoon. When AI-based weight estimation models are developed and applied within a single farm using standardized measurement timing stable and accurate performance can be achieved. However, constructing a single universal model intended for use across multiple farms with non-standardized measurement timing becomes inherently challenging. This is because time-of-day-dependent physiological weight fluctuations under non-standardized measurement timing introduce systematic, rather than random, variability into the training data, potentially leading to unstable model behavior and reduced generalizability.

A key implication of this work is that the evaluation framework itself, rather than the sensing technology, requires reconsideration. Treating instantaneous scale measurements as ground truth implicitly assumes that live weight is stable, an assumption that is physiologically incorrect. More meaningful evaluation strategies may involve comparing repeated measurements over time, assessing consistency across measurement modalities, or distinguishing between body mass and transient internal mass components. From this perspective, camera-based systems may provide complementary information rather than serving as imperfect substitutes for conventional scales.

The present study is intentionally theoretical and model-based. Our objective was not to validate a specific device or algorithm, but to clarify a fundamental limitation in how accuracy is currently defined and assessed for animal weight measurements. By integrating published behavioral and physiological data into a unified framework, we provide a quantitative basis for interpreting measurement discrepancies that are frequently reported but poorly explained. While empirical studies combining high-frequency camera-based measurements and continuous scale recordings would further strengthen this interpretation, the physiological constraints identified here are general and not dependent on any particular sensing technology.

Future work should first quantify within-day live-weight variation empirically under typical farm routines (including different feeding/drinking schedules and measurement times). These data should then be used to establish biologically meaningful reference protocols for studies so that technology ‘accuracy’ is not confounded by physiological dynamics. Such baselines will also support cross-farm studies by explicitly accounting for time-of-day-dependent variability in reference weights.

Overall, this study emphasizes that live weight should be understood as a dynamic physiological variable. Recognizing this property is essential for avoiding misinterpretation of measurement discrepancies and for developing evaluation criteria that are consistent with biological reality. Incorporating physiological variability into assessment frameworks will be critical for the responsible adoption and comparison of camera-based weight estimation technologies in pig production.

In commercial practice, body weight is typically measured using floor scales at discrete time points, often without strict control of measurement timing relative to feeding or drinking events. Repeated measurements within short time intervals are rarely performed, and short-term physiological body mass fluctuations are generally not explicitly accounted for in routine management decisions.

## 5. Conclusions

This study demonstrates that live weight in finishing pigs is an inherently dynamic physiological variable rather than a fixed reference value. Normal daily behaviors, including drinking, feeding, urination, and defecation, generate short-term weight fluctuations of approximately ±3–5 kg, even when measurement error is minimal. Therefore, the study goal was achieved by quantifying the expected magnitude of within-day physiological live-weight variation in finishing pigs, providing a reference for interpreting discrepancies between weighing technologies.

As a result, discrepancies of several kilograms between camera-based estimates and instantaneous scale measurements should not automatically be interpreted as inaccuracies. Instead, they reflect fundamental differences between body mass inferred from external morphology, which is less sensitive to transient internal components, and the instantaneous total mass measured by floor scales.

These findings indicate that commonly used accuracy metrics based on instantaneous scale readings may be insufficient when interpreted without considering the temporal dynamics of physiological weight variation. In particular, while accurate camera-based weight estimation models can be developed using standardized measurement timing within a single farm, the construction of a universal model applicable across farms with non-standardized measurement timing is fundamentally constrained by time-of-day-dependent physiological weight fluctuations under non-standardized measurement timing.

Recognizing the dynamic nature of live weight is therefore essential for developing biologically meaningful evaluation frameworks and for the appropriate interpretation and comparison of weight measurement technologies in pig production.

Accordingly, evaluation frameworks based on standardized measurement timing, repeated measurements, or averaged benchmarks may provide more biologically meaningful references than single instantaneous scale readings when assessing weight measurement technologies.

## Data Availability

The data supporting the findings of this study are available in the Supplementary Materials (Table S1 and Supplementary Methods S1). The simulation code used to generate the results is available from the corresponding author upon reasonable request.

## Supplementary Materials

The following supporting information can be downloaded at: https://www.mdpi.com/article/10.3390/ani16030498/s1, Table S1: Summary of model parameters used in the stochastic simulation of within-day live-weight dynamics in finishing pigs. The table lists distribution types, numerical ranges, fixed constants, and simplified dynamical assumptions (clearance times, transit times, and accumulation models), together with corresponding literature sources and physiological justification; Figure S1: One-factor-at-a-time sensitivity analysis showing the distribution of within-day live-weight fluctu-ation range across 1,000 simulated pigs for each parameter setting. Only one parameter was varied at a time, while all other baseline parameters were fixed as listed in Table S1.
