# Supplementary material for: Why Camera-Based and Scale-Based Measurements Differ: A Physiological Model of Diurnal Weight Variation in Finishing Pigs
Source: Animals (Basel). 2026 Feb 5;16(3):498. doi: 10.3390/ani16030498 (PMC12896642; doi:10.3390/ani16030498)
Supplement: Supplementary file 1 [file animals-16-00498-s001.zip › animals-4109460-supplementary.pdf]

Supplementary Materials

This document provides additional methodological details and parameter definitions supporting the main text.

Table S1. Summary of model parameters used in the stochastic simulation of within-day live-weight dynamics in finishing pigs. The table lists distribution types, numerical ranges, fixed constants, and simplified dynamical assumptions (clearance times, transit times, and accumulation models), together with corresponding literature sources and physiological justification.

Category	Parameter	Symbol	Distribution / Model	Value (code-defined)	Notes / Justification
**General**	Baseline body weight	(W_0)	Fixed	**100 kg**	Finishing pig; conservative lower-bound
	Time step	–	Fixed	**1 min**	Numerical resolution
	Simulation duration	–	Fixed	**24 h (1440 min)**	One diurnal cycle
	Net daily growth	–	Linear constraint	+**0.8 kg/day**	Typical finishing growth rate
**Drinking**	Drinking events per day	(N_d)	Poisson, clipped	mean 12 → **8–15 /day**	Drinking visit frequency
	Water mass per event	(M_d)	Gamma(3.0, 0.25), clipped	**0.4–1.6 kg/event** (mean ≈ 0.75)	Typical drinking bout size
	Event timing	–	Weighted sampling	Daytime (06:00–22:00) favored	Reduced night activity
	Water clearance	–	Linear decay	**180 min (3 h)**	Simplified clearance window
**Feeding**	Feeding events per day	(N_f)	Discrete uniform	**3–6 /day**	Typical meal structure
	Daily feed intake	–	Uniform	**2.5–3.0 kg/day**	Finishing pigs
	Meal size allocation	–	Dirichlet	Sum = daily intake	Preserves intra-day variability
	GI mass factor	–	Multiplicative	×**1.15**	Feed + bound water
	GI transit time	–	Linear decay	**12 h (720 min)**	Simplified GI passage
**Urination**	Urination events per day	(N_u)	Poisson, clipped	**6–10 /day**	Typical urination frequency
	Urine mass per event	(M_u)	Uniform	**0.5–1.5 kg/event**	Daily total defined by sum
	Urine model	–	Reservoir + emptying	Constant accumulation + discrete removal	Stepwise weight drops
**Defecation**	Defecation events per day	(N_e)	Discrete uniform	**2–4 /day**	Typical defecation frequency
	Feces mass per event	(M_e)	Uniform	**0.15–0.30 kg/event**	Daily total defined by sum
	Feces model	–	Reservoir + emptying	Constant accumulation + discrete removal	Stepwise weight drops

Supplementary Methods S1

To assess the robustness of the reported within-day live-weight fluctuation magnitude, one factor at a time (OAT) sensitivity analysis was conducted.

All baseline model parameters were fixed to the values listed in **Table S1**, and only a single parameter was varied at a time. The following three parameters, which directly reflect simplified modeling assumptions, were examined:

1. **Drinking-related mass clearance time**:
   120, 180 (baseline), and 240 min
2. **Gastrointestinal transit time**:
   10, 12 (baseline), and 14 h
3. **Urine mass per event**:
   0.5–1.0, 0.5–1.5 (baseline), and 1.0–2.0 kg per event

For each parameter setting, **1,000 simulated pigs** were generated using identical stochastic rules for all other parameters. For each simulated individual, the **within-day live-weight fluctuation range**, defined as the difference between the maximum and minimum body weight over a 24 h period, was calculated.

This analysis was designed to evaluate whether the magnitude of short-term live-weight variation is qualitatively sensitive to the choice of simplified clearance, transit, and excretion assumptions, rather than to provide an exhaustive parameter sweep.

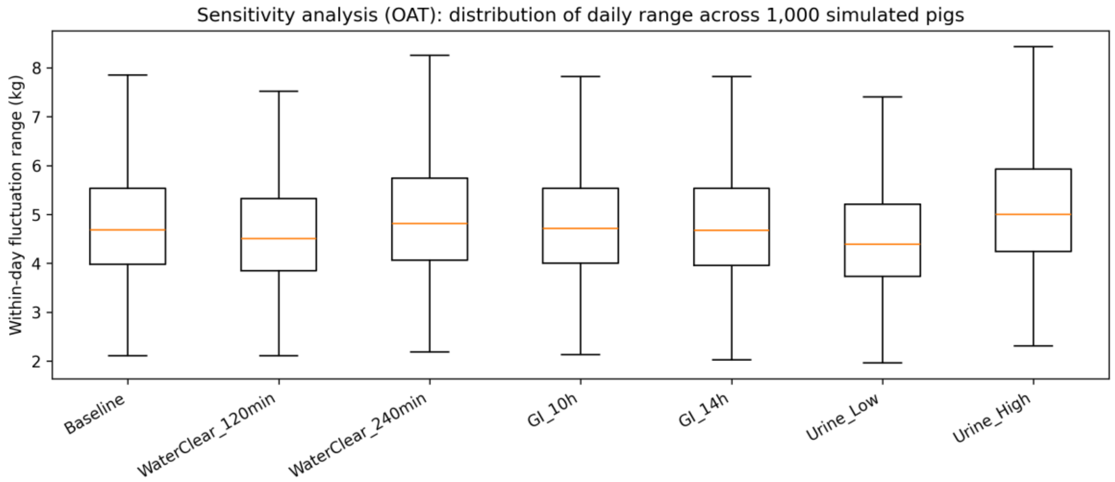

Figure S1. One-factor-at-a-time sensitivity analysis showing the distribution of within-day live-weight fluctuation range across 1,000 simulated pigs for each parameter setting. Only one parameter was varied at a time, while all other baseline parameters were fixed as listed in Table S1.
